# Application of biclustering of gene expression data and gene set enrichment analysis methods to identify potentially disease causing nanomaterials

**DOI:** 10.3762/bjnano.6.252

**Published:** 2015-12-21

**Authors:** Andrew Williams, Sabina Halappanavar

**Affiliations:** 1 Environmental Health Science and Research Bureau, Environmental and Radiation Health Sciences Directorate, Health Canada, Ottawa K1A 0K9, Canada

**Keywords:** gene expression, risk assessment, toxicogenomics

## Abstract

**Background:** The presence of diverse types of nanomaterials (NMs) in commerce is growing at an exponential pace. As a result, human exposure to these materials in the environment is inevitable, necessitating the need for rapid and reliable toxicity testing methods to accurately assess the potential hazards associated with NMs. In this study, we applied biclustering and gene set enrichment analysis methods to derive essential features of altered lung transcriptome following exposure to NMs that are associated with lung-specific diseases. Several datasets from public microarray repositories describing pulmonary diseases in mouse models following exposure to a variety of substances were examined and functionally related biclusters of genes showing similar expression profiles were identified. The identified biclusters were then used to conduct a gene set enrichment analysis on pulmonary gene expression profiles derived from mice exposed to nano-titanium dioxide (nano-TiO_2_), carbon black (CB) or carbon nanotubes (CNTs) to determine the disease significance of these data-driven gene sets.

**Results:** Biclusters representing inflammation (chemokine activity), DNA binding, cell cycle, apoptosis, reactive oxygen species (ROS) and fibrosis processes were identified. All of the NM studies were significant with respect to the bicluster related to chemokine activity (DAVID; FDR p-value = 0.032). The bicluster related to pulmonary fibrosis was enriched in studies where toxicity induced by CNT and CB studies was investigated, suggesting the potential for these materials to induce lung fibrosis. The pro-fibrogenic potential of CNTs is well established. Although CB has not been shown to induce fibrosis, it induces stronger inflammatory, oxidative stress and DNA damage responses than nano-TiO_2_ particles.

**Conclusion:** The results of the analysis correctly identified all NMs to be inflammogenic and only CB and CNTs as potentially fibrogenic. In addition to identifying several previously defined, functionally relevant gene sets, the present study also identified two novel genes sets: a gene set associated with pulmonary fibrosis and a gene set associated with ROS, underlining the advantage of using a data-driven approach to identify novel, functionally related gene sets. The results can be used in future gene set enrichment analysis studies involving NMs or as features for clustering and classifying NMs of diverse properties.

## Introduction

Metadata analysis that leverages genomics data has become increasingly popular as more experiments populate publicly available data repositories such as the Gene Expression Omnibus (GEO; http://www.ncbi.nlm.nih.gov/geo/) and European Bioinformatics Institute (EBI; https://www.ebi.ac.uk/arrayexpress/). A systems biology approach through meta-analysis has the potential to reveal relationships and insight on resulting phenotypes that may not be possible to detect through the analysis of any individual experiment [1–12].

Conventional molecular approaches for the study of organismal response to toxicant exposures or diseases involve the study of one gene or a few genes at a time, whereas biological response is driven by a group of genes. Thus, when normal function of a specific biological process is perturbed, alterations and enrichment in the expression of a subset of co-functioning genes associated with that biological process are observed. Toxicogenomic tools such as gene expression profiling have become a widely used strategy for investigating the genome-wide changes relating to molecular mechanisms underlying many complex responses and diseases. The fact that genes interact with each other and are expressed in functionally relevant patterns implies that gene-expression data can be grouped into functionally meaningful gene sets across a subset of conditions [13–32]. The analysis of such predefined gene sets is a powerful alternative to individual gene analysis [13]. However, derivation of meaningful and relevant gene sets from the thousands of genes showing expression changes following exposure to toxicants is challenging.

Gene set data analysis, a computational technique which determines if a predefined set of genes exhibit statistically significant differential expression between two or more experimental conditions (time, dose, tissue, etc.), relies on the knowledge of annotated pathways relevant to the underlying physiology or biology being investigated. A survey conducted by Huang et al. [33] identified 68 different gene set enrichment tools. These methods are applied to manually and computationally curated [29] gene sets to identify enriched functional groupings of genes. These gene set enrichment tools include DAVID [21–22], EASE [34], GoMiner [35], MAPPFinder [36], Onto-express [37] and others, which consist of controlled descriptions of gene functions that are frequently used to define gene sets. Other tools, such as pathway databases including Gene Ontology [38], KEGG [39], BioCyc [40], TfactS [41], CTD [42], and BioCarta (http://www.biocarta.com), have also been applied in gene set analysis. Despite the number of tools available, the effective identification of functional groups of genes relevant to the underlying physiology across several conditions still remains a challenge. As a result, these tools continue to be refined and improved.

Nanomaterials (NMs) are materials manufactured on the nanoscale (1–100 nm) and are the building blocks of nanotechnology. On the nanoscale, materials exhibit unique size-associated properties (optical, magnetic, mechanical, thermodynamic, electrical, etc.), which are harnessed for use in various commercial applications [43]. Current applications of NMs include therapeutic applications (e.g., nanomedicine, drug delivery, diagnostics), agriculture, manufacturing, electronics, cosmetics, textiles, and environmental remediation and protection. Although NMs are synthesized from their corresponding, known, bulk chemical substances, owing to their distinct size-associated properties, their biological or toxicological behavior are often different from their analogous bulk compound. Because of their smaller size and large surface area, NMs are known to have increased ability to interact with cellular membranes, they can easily cross cellular barriers and penetrate deeper regions of tissue (such as the highly vascularized alveolar regions of lungs), and they exhibit increased toxicological activity as compared to the corresponding bulk material or comparatively large particles [43]. A variety of conventional toxicology tools have been assessed using both in vitro and in vivo models for their suitability and applicability for toxicity testing of NMs. However, these tools are single-endpoint-based or targeted in nature, investigate only one type of response at a time, and lack detailed mechanistic information [44]. Given the rate at which nanotechnology is growing, and the limitations of the currently available toxicological testing tools, it is estimated that it will take several decades and millions of dollars to complete the assessment of NMs of various sizes, shapes and surface coatings that require immediate assessment [45]. Therefore, more efficient toxicity testing and prediction tools are needed to provide a comprehensive overview of the biological activities of NMs to rapidly screen the toxicological potential of NMs.

Over the last few years, genome-wide expression analysis tools have been used as an alternative approach to comprehensively investigate the toxicological response induced by various classes of NMs and to identify the properties of NMs that are responsible for eliciting adverse effects. We have previously used transcriptomics profiling tools to investigate the underlying mechanisms of toxicity induced by nanoparticles of titanium dioxide (nano-TiO_2_) [46–48] and carbon nanotubes (CNTs) [49–50] of various sizes and properties. This work identified the properties of nano-TiO_2_ that influence their inflammogenic potential [51]. These studies have generated a large repository of gene expression data that reflect the diversity of NM-induced biological response across a variety of experimental conditions. However, the challenge lies in the effective use of these data to discern individual or networks of genes conferring adverse outcomes of regulatory importance or disease phenotypes.

In the present study, we used a meta-analysis approach like that described by Turcan et al. [20] to identify functionally related biclusters of genes showing similar expression profiles, derived from publicly available gene expression data sets describing specific lung diseases (Table 1). One advantage of biclustering is that genes in the same cluster do not have to behave similarly over all experimental conditions. Unlike classical clustering techniques, biclusters can overlap with each other. This is ideal for mining functionally related gene sets as genes can be associated with more than one biological process. Several studies [3,52–55] have shown that biclustering is a useful methodology to uncover processes that are active only over some but not all experimental conditions [56].

**Table 1:** Publically available datasets.

	GEO accession; reference	Platform	Disease model/nanomaterial

Lung disease models	GSE4231 [57]	UCSF 10Mm Mouse v.2 Oligo Array (GPL1089); UCSF GS Operon Mouse v.2 Oligo Array (GPL3330); UCSF 11Mm Mouse v.2 Oligo Array (GPL3331); UCSF 7Mm Mouse v.2 Oligo Array (GPL3359)	Lung inflammation models
	GSE6116 [58]	Affymetrix Mouse Genome 430 2.0 Array (GPL1261)	Biomarkers to predict female mouse lung tumors
	GSE6858 [59]	Affymetrix Mouse Genome 430 2.0 Array (GPL1261)	Model of experimental asthma
	GSE8790 [60]	Affymetrix Mouse Genome 430 2.0 Array (GPL1261)	Cigarette smoke-induced emphysema
	GSE11037 [11]	Agilent-011978 Mouse Microarray G4121A (GPL891)	Emphysema
	GSE18534 [61]	Affymetrix Mouse Genome 430 2.0 Array (GPL1261)	Mouse small cell lung cancer model
	GSE19605 [62]	Illumina MouseRef-8 v2.0 expression beadchip (GPL6885)	Lung carcinogenesis
	GSE25640 [63]	Affymetrix Mouse Genome 430 2.0 Array (GPL1261)	Pulmonary fibrosis
	GSE31013 [64]	Affymetrix Mouse Genome 430 2.0 Array (GPL1261)	Spontaneous lung tumors
	GSE40151 [65]	Affymetrix Mouse Genome 430 2.0 Array (GPL1261)	Idiopathic pulmonary fibrosis
	GSE42233 [66]	Illumina Mouse WG-6 v2.0 expression beadchip (GPL6887)	Lung cancer
	GSE52509 [67]	Illumina MouseRef-8 v2.0 expression beadchip (GPL6885)	COPD

NM studies	GSE29042 [68]	GPL4134 Agilent-014868 Whole Mouse Genome Microarray 4x44K G4122F	CNT: MWCNT-7
	GSE35193 [48]	GPL7202 Agilent-014868 Whole Mouse Genome Microarray 4x44K G4122F	CB: Printex 90
	GSE41041 [47]	GPL7202 Agilent-014868 Whole Mouse Genome Microarray 4x44K G4122F	TiO_2_: UV-Titan L181
	GSE47000 [49]	GPL10787 Agilent-028005 SurePrint G3 Mouse GE 8x60K Microarray	CNT: Mitsui7
	GSE60801 [51]	GPL7202 Agilent-014868 Whole Mouse Genome Microarray 4x44K G4122F	TiO_2_: NRCWE-025, NRCWE-030
	GSE60801 [51]	GPL7202 Agilent-014868 Whole Mouse Genome Microarray 4x44K G4122F	TiO_2_ Sanding dust: Indoor-R, Indoornano TiO_2_
	GSE60801 [51]	GPL7202 Agilent-014868 Whole Mouse Genome Microarray 4x44K G4122F	TiO_2_: Sanding dust NRCWE-032, sanding dust NRCWE-033
	GSE60801 [51]	GPL7202 Agilent-014868 Whole Mouse Genome Microarray 4x44K G4122F	TiO_2_: NRCWE 001 (no charge), NRCWE 002 (positively charged)
	GSE61366 [50]	GPL10787 Agilent-028005 SurePrint G3 Mouse GE 8x60K Microarray	CNT: NRCWE-26, NM-401

In this study, experiments investigating lung diseases (including lung inflammation, emphysema, chronic obstructive pulmonary disease (COPD) or lung cancer) in mice using the whole genome gene expression tools were obtained from GEO. For each study, raw data were downloaded from GEO and normalized as described in the methods below. Biological replicates for each of the experimental conditions were averaged. All studies were merged together and biclustering was employed. Through this analysis, ten biclusters representing ten functional gene sets were identified. Using DAVID [21–22], the biological functions associated with these biclusters were identified. Next, we applied these candidate gene sets/biclusters to nine, publically available, toxicogenomic gene expression studies (Table 1, published studies from our laboratory) to examine the toxicity induced by a variety of NMs (nano-TiO_2_, CB and CNTs) to determine the disease significance of the altered gene expression profiles following exposure to NMs. The analysis was restricted to lung disease models since pulmonary response following NM exposure is well characterized.

## Results and Discussion

### Identification of biclusters of genes from lung disease models

**Figure 1:**
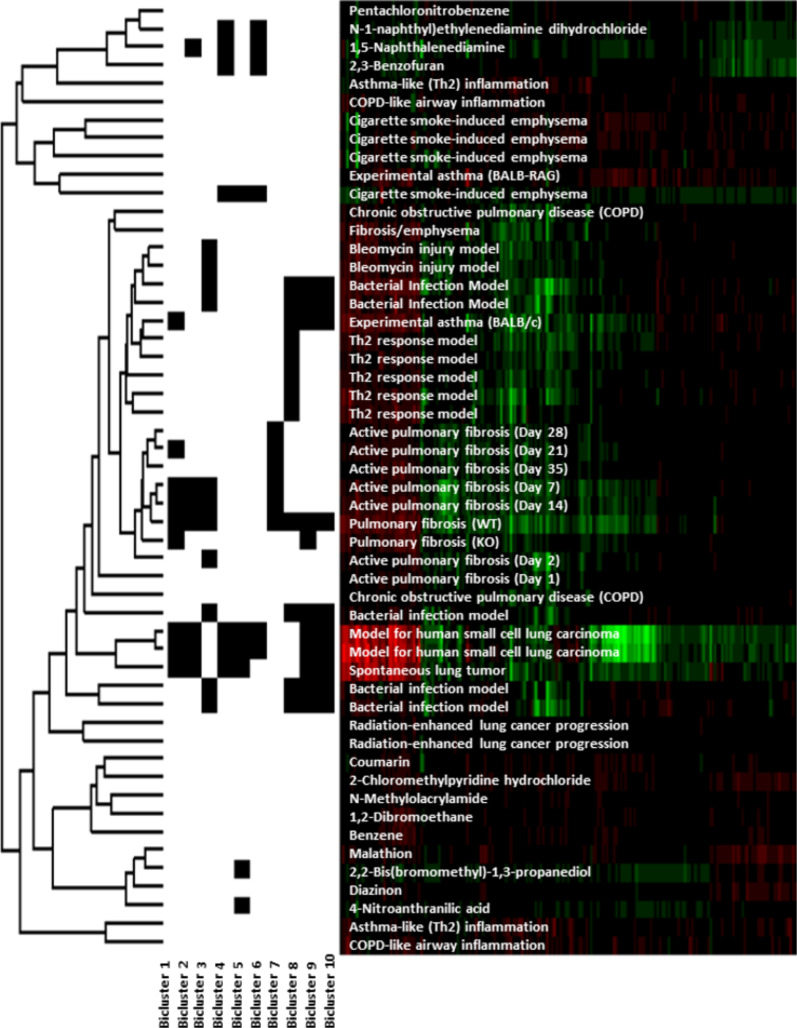
Heatmap of the gene symbols obtained from the bicluster data analysis. The distance metric used for the cluster analysis was 1-correlation estimated using Spearman correlation with average linkage.

To develop a data-driven view of the mouse lung response following exposure to NMs, publicly available genomic data from GEO that describe characteristic features of select lung diseases were leveraged. Eleven studies encompassing 52 experimental conditions with 8752 common gene symbols were assembled and specific gene sets were extracted using the repeated Bimax [69] biclustering method. A total of ten distinct biclusters were identified. The results of the biclustering are visually summarized in Figure 1.

Bicluster-1 consisted of studies investigating small cell lung carcinoma, spontaneous lung tumor, asthma and pulmonary fibrosis. This bicluster consisted of 19 gene symbols (C1qa, C3ar1, Cd68, Clec4n, Ctsk, Ect2, Fcgr3, Gp2, Igf1, Mmp12, Ms4a6d, Ms4a7, Pbk, Prc1, Saa3, Shcbp1, Spp1, Timp1 and Ube2c). Submitting these gene symbols into the DAVID functional annotation analysis tool (http://david.abcc.ncifcrf.gov) resulted in no significant gene ontology (GO). The top three ranked GO terms based on unadjusted p-values were acute inflammatory response (p-value = 0.0023), extracellular region (p-value = 0.0067) and extracellular region part (p-value = 0.0083). The lung disease models that comprised this bicluster were the model for human small cell lung carcinoma (GSE18534), spontaneous lung tumor (GSE31013), experimental asthma (GSE6858), active pulmonary fibrosis days 7, 14, and 21 (GSE40151) and pulmonary fibrosis (GSE25640).

The second bicluster consisted of twenty gene symbols (4632434I11Rik, Ccna2, Ccnb1, Ccnb2, Cdc20, Cdca8, Cldn4, Hells, Kif22, Mad2l1, Megf10, Melk, Msr1, Mx1, Plk4, Psat1, Rad51, Rrm2, Sprr1a and Uhrf1) with lung disease models such as a model for human small cell lung carcinoma, spontaneous lung tumor, chemical-induced lung carcinogenesis model from GSE6116 (1,5-naphthalenediamine; NAPD) and pulmonary fibrosis. Using DAVID, many GOs and Kyoto Encyclopedia of Genes and Genomes (KEGG) pathways were found significant (FDR p-value < 0.05). Ten of the twenty gene symbols from this bicluster were elements of the cell cycle GO (FDR p-value = 4.9 × 10^−6^) and five were part of the KEGG pathway (FDR p-value = 2.5 × 10^−4^).

The bleomycin injury and the bacterial infection models (GSE4231), as well as lung disease models related to pulmonary fibrosis, constituted the third bicluster. This bicluster contained 17 gene symbols (Aif1, Ccl2, Ccl9, Ccr5, Cdkn1a, Chl1, Cxcl9, Cyp7b1, Ereg, Fcgr1, Mt2, Retnla, Sfn, Sfrp1, Slc26a4, Socs3, and Tnc). Nine of the seventeen gene symbols are part of the extracellular region GO (FDR p-value = 0.0056). Other significant GO terms included chemokine receptor binding (FDR p-value = 0.017), extracellular region part (FDR p-value = 0.021) and chemokine activity (FDR p-value = 0.032).

The fourth bicluster contained gene symbols associated with chromatin binding (Arid4b, Atrx, Cnot6, Ezh2, Glmn, Hif1a, Ncl, Npm1, Ofd1, Sdccag1, Ssb, Tfrc, Tpp2, Ttc3, Zfp386) (FDR p-value = 0.019). This bicluster contained lung disease models associated with chemical exposure to known lung carcinogens (NAPD, *N*-1-naphthyl)ethylenediamine dihydrochloride (NEDD), 2,3-benzofuran (BFUR)) (GSE6116), a model for human small cell lung carcinoma, spontaneous lung tumor and cigarette smoke-induced emphysema (GSE8790). Many of the gene symbols found in this bicluster are transcription factors involved in the gene expression regulation and are associated with one form of cancer or another.

The fifth bicluster consisted of 35 gene symbols (1700019G17Rik, Ap1m2, Arg1, Atic, Cdc6, Ckmt1, Cldn7, Ddit4, Fetub, Galnt2, Gatm, Grb7, H1f0, Hdac11, Ildr1, Mapk13, Mcm2, Mcm5, Mcm6, Mrps15, Nup50, Pgls, Plek2, Psmd8, Rbp4, Rfc4, Rgl3, Rrs1, Serpine1, Sh3yl1, Slc25a13, Slc39a11, Spata5, Tk1, and Tmprss4). The lung disease models that formed this bicluster included the model for human small cell lung carcinoma, spontaneous lung tumor, cigarette smoke-induced emphysema and two chemical exposures, 2,2-bis(bromomethyl)-1,3-propanediol (BBMP; lung carcinogen) and 4-nitroanthranilic acid (NAAC; which resulted in no observed tumors). DNA replication for the GO term (FDR p-value = 4.1 × 10^−3^) and KEGG pathway (FDR p-value = 4.1 × 10^−3^) were significant. The only other significant GO term was DNA replication initiation (FDR p-value = 0.028). A few genes showed association with matrix degradation, inflammation and energy metabolism.

The sixth bicluster consisted of models for human small cell lung carcinoma, cigarette smoke-induced emphysema and chemical exposures BFUR, NAPD and NEDD. DAVID annotation analysis of the 23 gene symbols (Atm, Baz1b, Bclaf1, Ccar1, Dek, Dhx9, Epb4.1l3, F5, Hgf, Kif5b, Mier1, Pgm2l1, Plcb4, Ppil4, Rabep1, Smc1a, Stk3, Syncrip, Tcerg1, Ugcg, Usp9x, Zfml, and Zfp292) showed that this group of genes was primarily involved in the acetylation process (FDR p-value = 0.0056). Many GO terms related to the regulation of apoptosis were present in the results obtained by DAVID analysis. However, these results were not statistically significant after the FDR adjustment.

The seventh bicluster contained lung disease models related to pulmonary fibrosis only. DAVID analysis of the gene symbols included in this bicluster (Ccl3, Cd200r1, Chodl, Clec5a, Col24a1, Cxcl10, Emr1, Fxyd4, Gpnmb, Havcr2, Igj, Il1rn, Mmp10, Slc37a2, Syt12, Tgm1, Tlr8, Trem2, Wfdc12, and Zranb3) showed association with pulmonary fibrosis but no significant gene sets were derived. This bicluster can potentially serve as a candidate gene set for pulmonary fibrosis.

The eighth bicluster consisted of models for bacterial infection, Th2 response (GSE4231), asthma (GSE6858) and pulmonary fibrosis (GSE25640) with sixteen gene symbols (C1qb, Ch25h, Clec4a2, Ctss, F7, Fcgr2b, Itgam, Itgb2, Lgmn, Lpxn, Ly86, S100a4, Serpina3g, Serpina3n, Slc7a2, and Tbxas1). These gene symbols resulted in three significant GOs: response to wounding (FDR p-value = 0.0037), defense response (FDR p-value = 0.0063) and inflammatory response (FDR p-value = 0.0045).

The ninth bicluster consisted of the down-regulated gene symbols (Actc1, Cfd, Ckm, Ckmt2, Cox7a1, Cox8b, Csrp3, Eno3, Fmo3, Myh6, Myl1, Myl7, Pln, Pon1, Smpx, Sult1d1, Tnnc1, and Tnni3) and included a bacterial infection model, a model for human small cell lung carcinoma, spontaneous lung tumor, an asthma model and a pulmonary fibrosis model. These gene symbols were significantly associated with KEGG pathway cardiac muscle contraction (FDR p-value < 0.0001) and GO terms such as myosin complex (FDR p-value = 0.02) and regulation of system process (FDR p-value = 0.0015).

The tenth bicluster resulting from the analysis of the genes that were 2-fold down-regulated consisted of lung inflammation and disease models such as the bacterial infection model, a model for human small cell lung carcinoma, the study on spontaneous lung tumor, an asthma model and pulmonary fibrosis. This bicluster consisted of seventeen gene symbols (Aldh3a1, Bmp6, Cyp1a1, Cyp4b1, Eng, Fmo1, Fmo2, Gpr155, Igfbp6, Mapt, Ndrg2, Omd, Pcolce2, Pgam2, Scube2, Slc7a10, and Tnxb). These genes were associated with a variety of functions including fatty acid metabolism; however, DAVID functional annotation analysis of these gene symbols resulted in no statistically significant results to known annotated gene sets. However, several of these genes are associated with reactive oxygen species (ROS), which may not be a well-established gene set.

### Application of biclusters to classify NM-induced lung response

**Figure 2:**
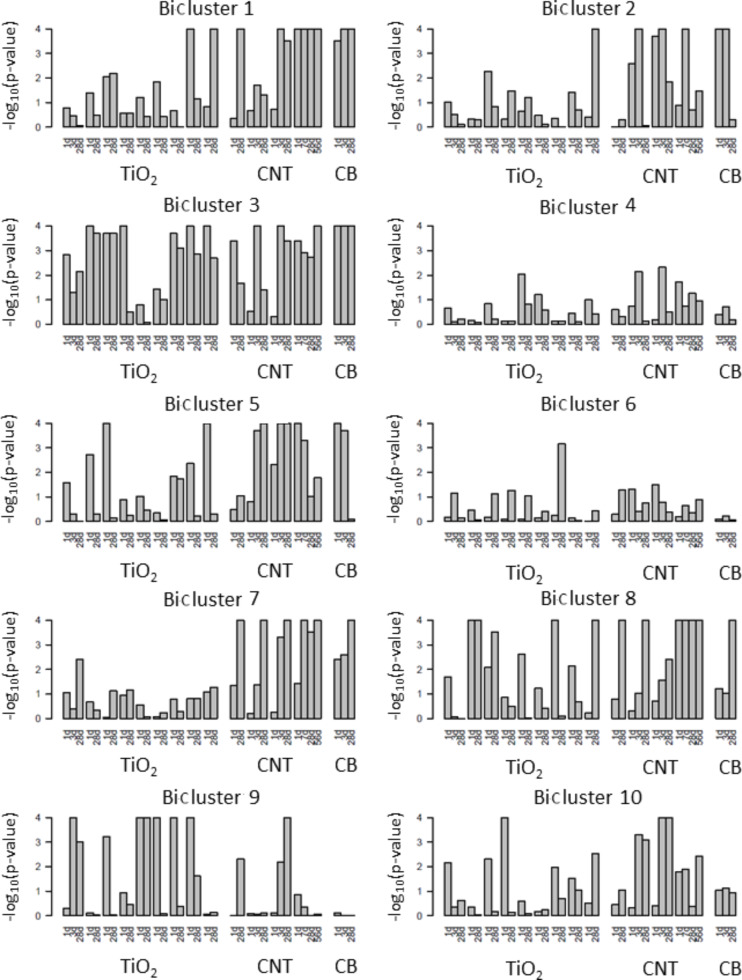
Gene set enrichment results of the NM datasets. Barplots of the −log_10_(p-value) from the GSEA are presented for each of the NM studies. The studies are ordered in the barplots as follows: TiO_2_: UV-Titan L181, NRCWE-025, NRCWE-030, Sanding Dust Indoor-R, Sanding Dust Indoornano, Sanding dust NRCWE-032, Sanding dust NRCWE-03, NRCWE 001 (No charge), NRCWE 002 (positively charged); CNT: Mitsui7, NRCWE-26, NM-401, MWCNT-7; CB: Printex 90.

Next, gene set enrichment analysis (GSEA) [29] using the bicluster-method-derived genes sets was conducted on the nine publically available studies [47–51,68] that examined NM-induced pulmonary toxicity. These results are presented in Figure 2. Bicluster-3 (genes associated with chemokine activity reflecting pulmonary inflammation) was enriched for most of the NMs. These results are in alignment with other studies in the literature that have shown pulmonary inflammation to be the predominant response following exposure to a variety of NMs. Bicluster-7 was the other significant cluster that was enriched in most of the experiments related to CNTs and CB. This cluster consisted of gene symbols showing strong association with pulmonary fibrosis. CNTs are well known to induce pulmonary fibrosis [50]. Although exposure to CB was not shown to cause lung fibrosis at the tested doses [48], studies have shown that CB exposure enhances bleomycin-induced lung fibrosis [70]. These results suggest that both carbon-based NMs may perturb similar biological processes and functions and factors in addition to the altered expression of a few genes in the gene set may contribute to the initiation of lung fibrosis.

## Conclusion

In this study, we examined the applicability of a data-driven approach to identify gene sets from the comprehensive gene expression data using a biclustering method. The results showed that the lung response to NM exposure predominantly reflects responses observed following bacterial infections and bleomycin injury models that involve acute inflammation. The combined biclustering and gene set enrichment analysis also identified CNT and CB as potentially fibrogenic NMs. Although several genes sets associated with acute DNA binding, cell cycle, apoptosis, and ROS response that were specific to different disease models were also observed to be perturbed following exposure to NMs, the implication of such perturbation was not clear from this analysis. In addition the identification of several previously defined, functionally relevant gene sets, the present study also identified two novel genes sets: Bicluster-7 (consisting of genes associated with pulmonary fibrosis) and Bicluster-10 (consisting of genes associated with ROS), underlining the advantage of using a data-driven approach to identify novel, functionally related gene sets. The results can be used in future gene set enrichment analysis studies involving NMs or as features for clustering and classifying NMs of diverse properties.

While powerful, data-driven meta-analysis approaches have several limitations. One important limitation is that the analysis is conditional on the subset of studies selected from the public data repositories such as GEO and EBI. Also, the experiments available in these repositories may not be representative of the population. For example, there are other mouse models of lung diseases that were not included in the present study due to lack of publicly available data or failure to meet the criteria set by the present study (time points, mouse strain, microarray platforms used).

The analysis is also limited to the gene symbols that were consistently investigated across the various microarray platforms from the different studies included in the analyses. Furthermore, the bicluster analysis is conditional to the two-fold change cut-off employed to create the binary matrix for the Bimax algorithm and the choice of the Bimax parameters. Modifying the fold cut-off to 1.75- and 1.5-fold, an additional 28 (23 up and 5 down) and 100 (89 up and 11 down) biclusters were identified. However, the interpretations were derived from the 2-fold cut-off as it provides the most conservative approach. The biclusters were stable when varying the minimum number of rows and when varying the minimum number of columns. Here, additional clusters were identified when these parameters were reduced and clusters were eliminated when these parameters were increased. Changes to any of the above could impact the final results and therefore the interpretation of the data.

## Experimental

### Lung disease models

The data were obtained from the GEO. The accession numbers for the studies [11,57–67] used in the exploration of novel gene sets are presented in Table 1. These data sets cover a variety of lung diseases and lung injury outcomes, including different lung inflammation models, emphysema, chronic obstructive pulmonary disease and experiments studying lung cancer and lung tumors. Several different microarray platforms including the Illumina expression beadchip were used in these studies. The analysis was restricted to lung disease models since pulmonary responses following exposure to NMs are well characterized.

### Data processing and normalization

The log_2_ transformation was applied to all signal intensity measurements. For the two color microarray studies, the LOWESS normalization method [71] using the R statistical software environment [72] was applied. For studies using the Affymetrix GeneChips^®^, the RMA normalization was applied using the justRMA function in the affy [73] R package. Quantile normalization was applied for studies that utilized the Illumina beadchip. This was done using the lumiN function in the lumi [74] R package.

Probes with technical replicates were then averaged using the median. The data for each study was then merged to its appropriate annotation file to obtain the gene symbol. Probes with the same gene symbol were then averaged using the median. The experimental conditions with biological replicates were averaged using the median. The median was used as it is a robust estimate of the central tendency.

For each experimental condition, the data was further normalized by centering to the matched control. The control samples were then removed from the data set. The remaining data is presented relative to the control, equivalently the log_2_ of the fold change (estimated using medians) for all the studies. The data were then merged across studies using the gene symbol. The mining the log_2_ of the fold changes was done in an attempt to minimize the cross-platform differences. However, platform differences may exist through compression of the fold-change values [75].

### Biclustering

The biclustering data analysis was conducted in R using the biclust [69] package. The repeated Bimax [56] method was selected for this analysis. Bimax uses a simple data model that assumes two possible states for each expression level, no change and change with respect to a control experiment. For this analysis, two binary matrices were constructed: one matrix, consisting of zeros and ones, where the ones indicated genes that were 2-fold up-regulated and a second matrix, where the ones identify genes that were 2-fold down-regulated.

The option for the minimum number of rows for the Bimax method was set at 15. The minimum number of columns (which represent the experimental conditions) was set as 5 and the maximum number of columns was set as 15. This resulted in 8 biclusters from the binary matrix representing the up-regulated genes and 2 biclusters were identified for the matrix representing the down-regulated genes.

### NM-induced lung response data sets

The data sets examining differential gene expression in mouse lung exposed to CB, nano-TiO_2_ or CNTs were compiled from GEO. Since this is a proof-of-concept study, the investigation was limited to those NMs for which lung toxicological response is well characterized. Also, the genomics datasets with multiple doses and post-exposure time points were considered in the analysis. The GEO accession numbers for these studies are presented in Table 1. These studies utilized the two color Agilent microarray reference design [76]. The data were LOWESS normalized and probes with technical replicates were averaged. The annotation file containing the gene symbol was merged with the expression data and probes with multiple gene symbols were averaged using the median expression.

### Gene set enrichment

As the NM-induced lung response data sets contained multiple doses, the test statistic from the Attract [19] approach was used. Using this method, the overall F-statistic for the dose effect was estimated for each gene. The F-statistics were then log_2_-transformed. A two sample t-test (assuming unequal variances) was then conducted, comparing the mean of the log_2_ F-statistics within the bicluster to the mean of the log_2_ F-statistics for all genes. The observed t-statistics and p-values are reported in Figure 2.
